# Strategies to improve quantitative assessment of immunohistochemical and immunofluorescent labelling

**DOI:** 10.1038/srep10607

**Published:** 2015-06-11

**Authors:** Sarah J. Johnson, F. Rohan Walker

**Affiliations:** 1 School of Electrical Engineering and Computer Science, University of Newcastle, Callaghan, NSW, Australia; 2 School of Biomedical Sciences and Pharmacy, University of Newcastle, Callaghan, NSW, Australia; 3 Hunter Medical Research Institute, New Lambton Heights, NSW, Australia

## Abstract

Binary image thresholding is the most commonly used technique to quantitatively examine changes in immunolabelled material. In this article we demonstrate that if implicit assumptions predicating this technique are not met then the resulting analysis and data interpretation can be incorrect. We then propose a transparent approach to image quantification that is straightforward to execute using currently available software and therefore can be readily and cost-effectively implemented.

At present, the most common approach for the quantitative assessment of images of immunohistochemical and immunofluorescent labelled material is an analysis technique commonly referred to as ‘thresholding’^1^,^2^,^3^,^4^,^5^,^6^. Essentially, an image acquired on a standard light, epi-fluorescent or confocal microscope is passed into an analysis program (e.g. Image-J, Fiji, Metamorph™, Imaris™ or equivalent) in which a particular pixel intensity level (the threshold) is manually defined and then used to demarcate what is considered to be ‘signal’ (the immunolabelled material of interest) and ‘noise’ (non-specific material attributable to the immunolabelling process). The number of pixels within the signal range is then quantified and compared across treatment groups.

Although no field-wide standards exist in biomedical science for quantification of immunolabelled material, it is widely accepted that a thresholding procedure can only provide genuinely valid results if certain assumptions concerning the immunolabelling and imaging processes are met. Broadly, it is recognised that all procedures must be completed under as close to identical conditions as is possible. For instance: (i) the same primary and secondary antibodies should be applied to all tissues, (ii) the same reagents should be used at the same concentrations (iii) and all incubation and development times should be identical. What is less frequently recognised is that valid thresholding also involves certain assumptions that are often non-explicit. If these implicit assumptions are not appropriately met, straightforward face-value interpretation of the analyses can become very challenging.

To better understand the nature of the implicit assumptions associated with the thresholding procedure it is useful to briefly describe the process that is employed to derive data from it. Typically, a user will take a set of images from a given experimental setup (involving two or more groups of images) and will adjust the threshold cut-point until the algorithm selects as signal a subset of the image they are ‘happiest’ with. The same threshold cut-point is applied to images from both groups and the amount of signal material compared across groups. In undertaking this approach the user is making a critical assumption, namely that the difference between groups is constant over the full set of what could be considered reasonable choices for the threshold (the threshold range). Critically, if the differences between groups across the signal spectrum are non-constant (small at some pixel intensities and large at others) a difference that may exist could be missed, and in the worst case scenario the set-point for thresholding could be manipulated in order to arbitrarily inflate or minimise relative group differences. Fig. 1 shows this effect using real experimental data. At the present time it is not straightforward to determine the extent to which these types of problems are inherent in the existing literature given the paucity of information conveyed when the results are reported for only a single threshold.

The fact that thresholding involves making a choice at one single level is in effect a historical artefact, emerging principally because the readily available software for undertaking the procedure has only allowed one thresholding level. This does not need to be the situation moving into the future. Indeed, the intensity information contained within an image can be readily extracted to examine differences across all pixel intensities rather than just one. Utilising all the information that is contained within an image (i.e. the pixel intensity histogram) has the distinct advantage of being able to visualise and quantify the degree of difference between groups across all thresholding levels. The data derived from this technique can be used to minimise the likelihood that a set-point for thresholding can be manipulated or be modified to inflate or minimise group differences.

The process of utilising all the available information within an image for the purpose of quantification begins by taking a standard grayscale image and creating a pixel intensity histogram. In the case of an 8 bit image this involves determining the number of pixels that occur at each of the 256 pixel intensities. This procedure is straightforward to execute in a package such a Fiji and is done by calling the ‘histogram’ function. The histogram can then be used to create a cumulative threshold spectra (CTS) by calculating what percentage of the total number of pixels in an image occur on or below each of the pixel intensities. We illustrate this process in Fig. 2. *The advantage of calculating the CTS is that it provides a plot of the percentage thresholded result for every possible threshold value and can be used to succinctly evaluate the extent of group differences through the entire threshold range rather than one arbitrary point.*

The pixel intensity histograms and the CTS can be used to used to complement the standard thresholding approach in two ways. Firstly the pixel intensity histograms and the CTS will be useful in preliminary studies to understand the effect of an intervention and to determine the robustness of any differences to the choice of threshold. Secondly, including the pixel intensity histograms and/or the cumulative threshold spectra when publishing thresholding results will provide a reader with information on the appropriateness of the chosen threshold by showing i) where the chosen threshold lies within the threshold range; ii) where in the threshold range the differences between groups occur; and iii) how much the group differences change across the signal range (extracted by the % differences plot). Information on how statisically representative the chosen threshold is, of all possible thresholds within the threshold range, can be derived by simply counting the fraction of thresholds within the threshold range that would yeild statistically significant diference had they been chosen. Furthermore, the pixel intensity histograms and/or the cumulative threshold spectra can be used to understand the source of any threshold difference (**the supplementary file contains a detailed and extensive explanation**).

Ideally, future efforts to quantify group differences in immunolabelled material will provide information on the pixel intensity histograms and/or cumulative threshold spectra to supplement any binary thresholding result. Providing the cumulative threshold spectra would allow those evaluating the results of a quantification procedure clearer access to the relative differences between groups using all the information available within the image, rather than the sliver of it chosen by the experimenter. This final data could then be presented alongside with a description of the degree to which group differences vary across the threshold range and how many of the pixel intensity levels within the threshold range achieve statistical significance. The net effect of this approach should be to allow both the investigator and the audience to have a much higher level of confidence in the end result of the analysis. Ultimately, wider adoption of this approach could provide for greater robustness of presented data and a more straightforward pathway towards data replication.

## Additional Information

**How to cite this article**: Johnson, S. J. and Walker, F. R. Strategies to improve quantitative assessment of immunohistochemical and immunofluorescent labelling. *Sci. Rep.* **5**, 10607; doi: 10.1038/srep10607 (2015).

## Figures and Tables

**Figure 1:**
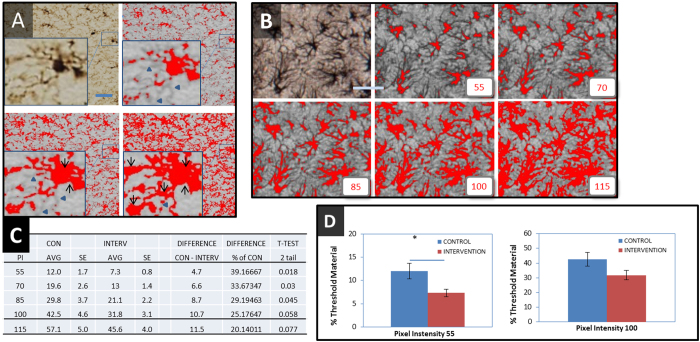
(**A**) Illustrates the standard thresholding procedure on a 20× image of Iba-1 labelled microglial cells from the lateral hypothalamus of the rat passed through a standard thresholding routine using the threshold function within Fiji. Upper right: using a relatively conservative inclusion criteria (included material is shown in red) substantial portions of the branching structure of the cell are omitted (as indicated by blue arrow heads). Lower left: increasing the level of threshold inclusiveness results in substantially more of the cell processes being included but some evidence of background inclusion (otherwise known as ‘flaring’) can be observed (black arrows). Lower right: increasing the threshold further results in complete coverage of the original cell by the threshold but results in substantial levels of flaring. (**B**–**D**) Examines the process of deploying conventional thresholding on a set of images from an actual experiment looking at whether or not an experimental intervention has modified the expression of a protein known as glial fibrillary acid protein (GFAP) within astrocyte cells in the hippocampus (n = 8/group). (**B**) Illustrates the thresholding procedure on a 20× image from a control showing the respective material included if the threshold cut-point was set at a pixel intensity of 55, 70, 85, 100 and 115 (out of 256 possible intensities). (**C**) The number of pixels included at each threshold (expressed as a % of the total number of pixels in the image) was calculated. The table shows: the average threshold amounts for each group; the raw difference between the groups; the between group difference expressed as a percentage of control; and the p-values for the group differences. (**D**) Illustrates how the column graphs would appear in a scientific report if a pixel intensity of 55 or 115 were chosen for the thresholding process. Two valid thresholding choices result in opposite scientific conclusions on the experimental intervention. Scale bar = 30 μm.

**Figure 2:**
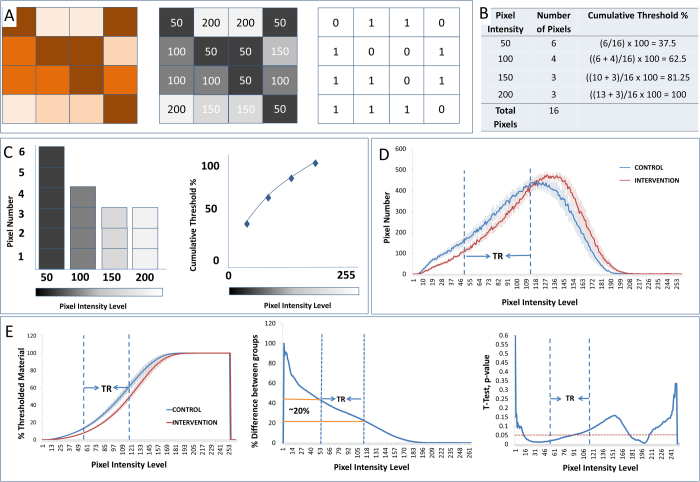
Illustrates the standard thresholding process and its adaption to create the cumulative threshold spectra. Panel (**A**) illustrates standard thresholding. A hypothetical 16 pixel 24 bit color image (left) is converted into an 8 bit greyscale image (middle). The greyscale image is thresholded at pixel intensity 50 to create a black (0) and white (1) binary image (right). Panels (**B**–**C**) illustrate the cumulative threshold spectra. Instead of simply determining the number of pixels at or below a single threshold the cumulative threshold spectra involves determining the amount of material included at each of the four possible thresholding cut-points. Panel (**B**) specifically illustrates the calculations used to create the histogram and cumulative threshold percentages. (**C**) Using the data presented in panel B, a pixel intensity histogram has been created (left) and the number of pixels occurring at each of the pixel intensities is presented graphically as a cumulative threshold spectra (right). Panel (**D**) represents the average pixel intensity histograms (±) SEM for an actual set of data representing the images as considered in Fig 1 derived from two groups of animals. (**E**) The left image illustrates the average cumulative threshold spectra (±) SEM for the control and intervention groups. The valid threshold range (TR, as identified in Fig. 1) is indicated by two vertical dashed lines bisecting the horizontal axis at pixel intensities 55 and 115. From the cumulative threshold spectra we can create a % difference plot, the middle image on panel E, which displays the same information in a different way. The % differences plot shows directly how a percent difference measure would vary as the threshold is varied. In the right most image of panel E is presented the probability values, which would have been derived from independent samples t-tests (2 tailed) for each of the 256 possible thresholding levels. The dotted red line again indicates the 0.05 significance level. In the case of our GFAP example we find that 36 of a total of 61 possible threshold levels within the valid threshold range are statistically significant (at the 0.05 level).
